# On the Post-Printing Heat Treatment of a Wire Arc Additively Manufactured ER70S Part

**DOI:** 10.3390/ma13122795

**Published:** 2020-06-21

**Authors:** Alireza Vahedi Nemani, Mahya Ghaffari, Ali Nasiri

**Affiliations:** Faculty of Engineering and Applied Science, Memorial University of Newfoundland, St. John’s, NL A1B 3X5, Canada; mghaffari@mun.ca (M.G.); amnasiri@mun.ca (A.N.)

**Keywords:** wire arc additive manufacturing, ER70S-6, anisotropic mechanical properties, microstructure, heat treatment

## Abstract

Wire arc additive manufacturing (WAAM) is known to induce a considerable microstructural inhomogeneity and anisotropy in mechanical properties, which can potentially be minimized by adopting appropriate post-printing heat treatment. In this paper, the effects of two heat treatment cycles, including hardening and normalizing on the microstructure and mechanical properties of a WAAM-fabricated low-carbon low-alloy steel (ER70S-6) are studied. The microstructure in the melt pools of the as-printed sample was found to contain a low volume fraction of lamellar pearlite formed along the grain boundaries of polygonal ferrite as the predominant micro-constituents. The grain coarsening in the heat affected zone (HAZ) was also detected at the periphery of each melt pool boundary, leading to a noticeable microstructural inhomogeneity in the as-fabricated sample. In order to modify the nonuniformity of the microstructure, a normalizing treatment was employed to promote a homogenous microstructure with uniform grain size throughout the melt pools and HAZs. Differently, the hardening treatment contributed to the formation of two non-equilibrium micro-constituents, i.e., acicular ferrite and bainite, primarily adjacent to the lamellar pearlite phase. The results of microhardness testing revealed that the normalizing treatment slightly decreases the microhardness of the sample; however, the formation of non-equilibrium phases during hardening process significantly increased the microhardness of the component. Tensile testing of the as-printed part in the building and deposition directions revealed an anisotropic ductility. Although normalizing treatment did not contribute to the tensile strength improvement of the component, it suppressed the observed anisotropy in ductility. On the contrary, the hardening treatment raised the tensile strength, but further intensified the anisotropic behavior of the component.

## 1. Introduction

Wire arc additive manufacturing (WAAM) is a novel technology capable of producing metallic components utilizing an arc welding process to additively fabricate engineering parts [1], with various applications such as impeller blades [2], bridge structures [3], shipbuilding plates [4], and wing ribs in the aerospace industry [5]. Different from the metal powder-based additive manufacturing processes, such as direct metal laser sintering (DMLS) and selective laser melting (SLM), wire arc additive manufacturing uses a consumable metallic wire as the feedstock material [6]. In WAAM, the entire consumable wire is continuously fed into an adopted electric arc or plasma, leading to an extremely high deposition rate as compared to that of the powder-based AM systems [7]. Therefore, wire-based systems are generally suitable for producing large-scale components with less complexity in geometry and design, in contrast to the powder-bed systems, which typically fabricate small and high-definition parts [8]. From another perspective, powder-bed additive manufacturing techniques are limited to a build envelope, but in wire-based systems, the torch is usually mounted on a robotic arm having more freedom of movement, implying that the component’s size is not confined by a chamber.

During wire arc additive manufacturing process, the feedstock material is melted and deposited in the form of weld beads layer by layer on the previously solidified tracks. As the consecutive layers fuse into the previous ones, the material is built up until the near-net-shape component is completed [9]. Since the process is involved with sequential melting and solidification, each region of the component is subjected to periodic fast heating and cooling cycles by the deposition of upper layers. Such complex localized thermal cycles lead to heterogeneous microstructure and anisotropic mechanical properties in the AM-fabricated components [1,10,11]. This is one of the main drawbacks of the WAAM technique as compared to the conventional methods of manufacturing. Sridharan et al. [12] studied the microstructure and mechanical properties of low-carbon low-alloy steel (ER70S) built through additive manufacturing and reported anisotropic elongation percentages in different directions. They concluded that the anisotropy in mechanical properties is related to the inhomogeneous and localized microstructure [12]. Haden et al. [13] also investigated wire arc additive manufacturing of 304 stainless steel and reported graded wear and hardness properties in both deposition and building directions. Their findings showed that this anisotropy is due to the fluctuation in localized thermal histories, consequently leading to the formation of a variety of microstructures, from austenitic to solidification structures owning mixed ferrite morphology with abrupt texture changes at different regions of the sample [13].

In addition to the microstructural inhomogeneity, the formation of internal defects between the deposited layers as a result of high heat removal capacity from the inter-pass regions may deteriorate the mechanical properties of the additively manufactured components [6,12,14,15]. The inter-pass defects being formed in the fusion boundaries commonly include entrapped gas, porosities, and lack of fusion [16,17]. The presence of the mentioned discontinuities acting as a stress riser in the structure can potentially make crack initiation sites leading to premature brittle fracture under tension, which has been extensively investigated in a previous authors’ publication [14].

Interestingly, the heterogeneous microstructure and anisotropic mechanical properties in a WAAM-fabricated part can be minimized by applying tailored post-printing heat treatment cycles [4]. For instance, Wang et al. [18] studied the effect of heat treatment on the anisotropic mechanical properties of a WAAM-fabricated H13 steel. They reported that the homogeneous microstructure achieved by annealing heat treatment for four hours at 830 °C led to diminishing the anisotropic mechanical properties of the part. Xu et al. [19] also successfully minimized the microstructural inhomogeneity of a wire arc additive manufactured maraging steel part by performing a post-process heat treatment i.e., solutionizing and aging, which resulted in a significant improvement in mechanical properties of the WAAM-fabricated component. Although applying a post-fabrication heat treatment has been previously reported by different studies [18,19,20], to the best of the authors’ knowledge, heat treatment of WAAM ER70S-6 has not been investigated heretofore, except for one of the authors’ previous studies [4], in which the impacts of a different heat treatment cycle (inter-critical austenitizing temperature) on the WAAM ER70S-6 microstructure and mechanical properties were investigated.

In this study, with the aim of homogenizing the microstructure and diminishing the induced anisotropy in an as-printed WAAM-ER70S-6 low-carbon low-alloy steel part, two heat treatment cycles, including normalizing (austenitizing followed by still-air cooling) and hardening (austenitizing followed by water quenching) from an upper-critical austenitizing temperature, were conducted on the as-printed samples. Microstructural and mechanical property characterizations were carried out on both as-printed and heat-treated samples in different orientations, including deposition (horizontal) and building (vertical) directions.

## 2. Materials and Methods

### 2.1. Material, Fabrication Process, and Post-Fabrication Heat Treatment

**Figure 1:**
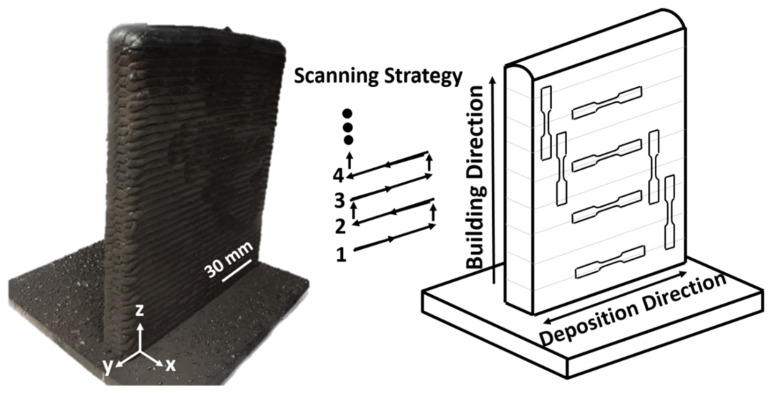
Schematic illustration of the wire arc additive manufacturing (WAAM) wall and tensile sample selection.

In the present study, a wall of low-carbon low-alloy steel (ER70S-6) was fabricated using the wire arc additive manufacturing method utilizing a Lincoln Electric GMA machine (Cleveland, OH, USA) with a torch mounted on a 6-axis Fanuc robot as the power source. To minimize the heat input of the WAAM process and be able to adjust the heat independent of the wire feed speed, an advanced current controlled surface tension transfer (STT) process was employed for fabrication. Utilizing the STT can further contribute to reducing the surface irregularities and spattering during the building process [21]. In order to smoothly feed the wire to the melt pool, the stand-off distance was held constant at 3 mm between the tip of the filler wire and the surface of the previous layer. Figure 1 schematically represents the set-up of the WAAM process.

Table 1 shows the nominal chemical composition of the ER70S-6 feedstock solid wire with 0.9 mm diameter manufactured by Lincoln Electric. The selected WAAM process parameters yielding the optimum bead quality and appearance with minimum spattering are listed in Table 2. ASTM A36 mild steel with a thickness of 12 mm was used as the substrate, which was attentively wire brushed and then cleaned by acetone to prevent contamination of the melt pools and the formation of gas pores during the solidification process. The whole part contained 50 consecutive layers, and each layer was comprised of six beads with a length of 135 mm and a 3 mm center-to-center overlap, resulting in a wall with a total width of 22 mm and a height of 150 mm. Employing a Thermo-Scientific Lindberg furnace, two heat treatment cycles were applied to the as-printed component, including (i) normalizing (austenitizing followed by still-air cooling), and (ii) hardening (austenitizing followed by water quenching). For initial austenitizing in both cycles, the samples were heated up to 900 °C for 1 h. The purpose of the normalizing process was to homogenize the microstructure by producing a uniform grain size along the melt pools, fusion boundaries, and heat affected zones. The intention of the hardening heat treatment was also to increase the hardness and tensile strength of the component.

### 2.2. Microstructural Characterization

To prepare the samples for microstructural characterizations, a Tegramin-30 Struers auto-grinder/polisher (Cleveland, OH, USA) was employed, then the samples were etched chemically using a 5 vol.% Nital reagent for 15–20 s [22]. The microstructure of the fabricated component was characterized at different regions from the bottom to the top of the wall to detect any microstructural changes throughout the whole part. To perform the microstructural characterization at different magnifications, an optical microscope (Nikon Eclipse 50i, Shinagawa, Tokyo, Japan) and a field emission scanning electron microscope (FEI MLA 650F, Hillsboro, OR, USA) were employed.

### 2.3. Mechanical Properties Evaluation

Microhardness distribution was measured and plotted along a line covering five successive layers through the building (vertical) direction on different zones including the center of the melt pools, fusion boundaries, and heat affected zones (HAZs), using a Buehler Micromet hardness test machine (Lake Bluff, IL, USA) with the applied load of 300 g and an indentation time of 45 s. It should be noted that the reported data of microhardness are the average of five different measurements. The indentations were done on the polished and etched surfaces in order to distinguish the position of each indentation relative to the melt pool’s geometry. Moreover, microhardness profiles were produced by subsequent indentations with 300 µm intervals (approximately five times more than the diagonal of each indent) to avoid the work hardening effect. 

Tensile test specimens from the as-printed and heat-treated samples were machined parallel and perpendicular to the building directions based on the ASTM E8m-04 standard sub-size specimen (West Conshohocken, PA, USA) [23] with dimensions of 100 mm × 25 mm × 5 mm. The room temperature uniaxial tensile tests were carried out using an Instron load frame (Norwood, MA, USA) equipped with an extensometer at the crosshead speed of 8 mm/min. Each tensile test was repeated five times under the same conditions to obtain a reliable average value. 

## 3. Results and Discussion

### 3.1. Microstructural Characterization

**Figure 2:**
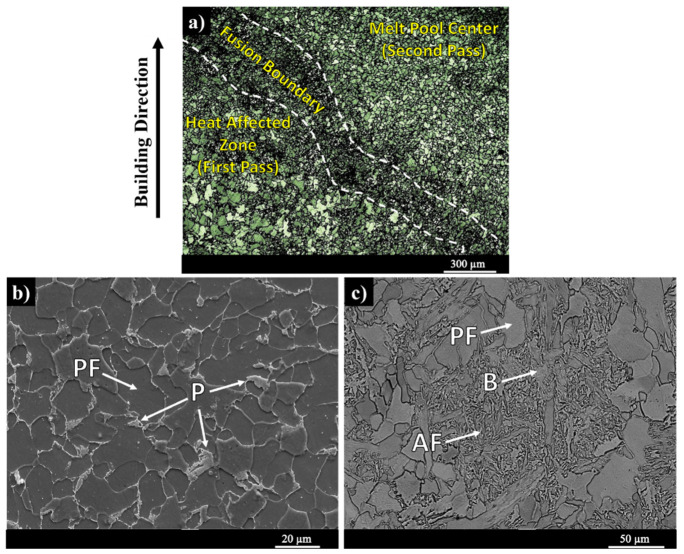
(**a)** Low magnification optical micrograph of the as-printed sample, (**b)** higher magnification SEM micrograph of the melt pool center, and (**c)** fusion boundary (PF: polygonal ferrite, P: lamellar pearlite, B: bainite, AF: acicular ferrite).

Figure 2a illustrates a low magnification OM micrograph of the as-printed sample showing the transition from the center of the melt pool to the melt pool boundary and then the heat affected zone. The dominant microstructure in the center of the melt pool consists of a low volume fraction of lamellar pearlite (P) primarily formed at the grain boundaries of polygonal ferrite (PF) (Figure 2b). Figure 2c depicts the SEM micrograph taken from the melt pool boundary region (denoted as the fusion boundary shown in Figure 2a) at higher magnification, revealing the formation of acicular ferrite (AF) and bainite (B) due to the faster cooling rate along the boundaries of each deposited bead as compared to its center. The aforementioned transition in the microstructure during 3D-printing of ER70S wire is also reported by Haselhuhn et al. [24]. In another investigation, Lee et al. [25] also studied the microstructure of the welded low-carbon low-alloy AH36 steel and similarly reported the formation of acicular ferrite and bainite near the fusion line.

It is well established that the presence of acicular ferrite and bainite constituents in the microstructure of steels can promote the mechanical properties of the component. This is primarily resulted from the finer structure of both phases, a more uniform distribution of carbide and higher dislocation density and internal stresses in the bainite phase, contributing to a higher hardness/strength and ductility in the alloy [26,27,28]. However, it should be noted that since the volume fraction of acicular ferrite and bainite constituents are negligible as compared to the dominant ferritic and pearlitic microstructure of the alloy, the presence of acicular ferrite and bainite cannot have a significant contribution to the mechanical properties of the WAAM-ER70S sample. On the other hand, according to Figure 2a, the microstructure of the HAZ consists of coarser grains of polygonal ferrite in comparison with the interior of the melt pool, as the thermal cycle associated with each depositing track facilitates the grain growth in the previous bead. The grain coarsening in the HAZ can potentially lead to a remarkable softening in this area, consequently resulting in a reduced localized strength and hardness in a sample that accommodates this region [29]. The formation of such microstructural inhomogeneity from the center of the melt pool to its boundaries and to HAZ is attributed to the overlapping scanning strategy associated with the multi-layer deposition nature of the WAAM process. Consequently, this process evokes various thermal cycles in different regions of the sample [30].

**Figure 3:**
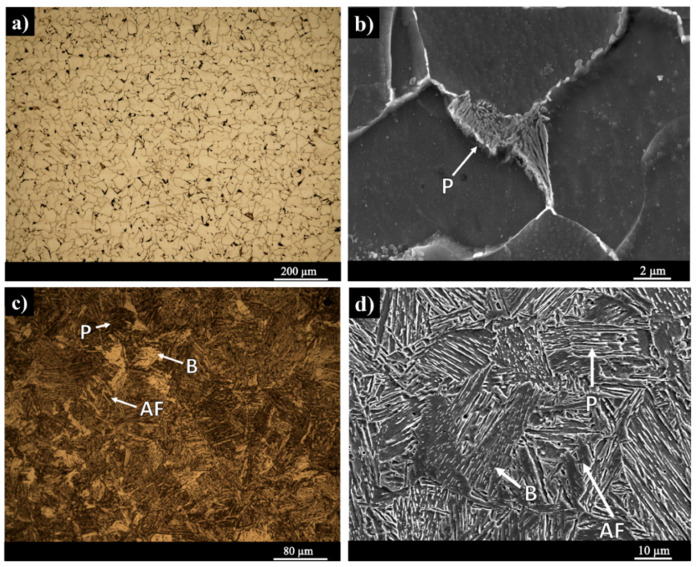
The microstructure of the normalized sample (**a**,**b**), and hardened (water-quenched) sample (**c**,**d**) at different magnifications.

Post printing heat treatment is commonly used to modify the microstructure and, consequently the mechanical properties of an additively manufactured component [4,20]. Figure 3a–d depict the microstructure of the WAAM-ER70S-6 wall after applying different heat treatment cycles, including normalizing (Figure 3a,b) and hardening (Figure 3c,d) at different magnifications. According to the thermodynamically simulated continuous cooling transformation (CCT) diagram for the ER70S-6 wire [31], moderate cooling rates in the range of 10–100 °C/s results in a ferritic–pearlitic microstructure, while severe cooling rates in the range of 0.1–1 °C/s leads to the formation of non-equilibrium phases such as bainite. As indicated in Figure 3a,b, the normalizing heat treatment with moderate cooling rate from the austenitizing temperature of 900 °C, has not altered the pre-existing constituents of the microstructure of the as-printed sample. However, the grain size became more uniform and homogenous from the center of each melt pool toward the heat affected zone in the adjacent track. In other words, the grain coarsening in the heat affected zone was eliminated after normalizing heat treatment.

For the initial austenitizing step, the sample was heated up to 900 °C, where γ is the only stable phase since the *Ac_3_* temperature of the alloy was calculated to be at ~883 °C, using a reported empirical equation that predicts austenite formation temperatures, i.e., *Ac_1_* and *Ac_3_*, for the low-alloy steels with less than 0.6 wt.% C [32]. Subsequently, uniformly distributed austenite grains nucleate and grow evenly in any region of the material during austenitization. Following the full austenitization of the microstructure, the sample is subjected to a relatively slow cooling by exposing the sample to room temperature. The slow cooling rate associated with the normalizing heat treatment hinders the formation of unstable or metastable phases, such as bainite or martensite, during the normalizing heat treatment. Therefore, the microstructure of the sample in terms of the formed constituents was analogous to the dominant microstructure of the as-printed sample containing polygonal ferrite, pearlite and precipitation of tertiary cementite, whilst the grains obtained a more homogeneous and uniform distribution after the normalizing heat treatment. The precipitation of the tertiary cementite in the ferrite grain boundaries, as a high energy site for nucleation of a new phase, has been also reported in other grades of low-carbon steels family [33,34]. Natividad et al. [35] also performed the normalizing heat treatment on Grade X70 pipeline steel and reported the formation of polygonal ferrite and pearlite areas with more homogenous and uniform microstructure in comparison with the as-received material. Figure 3c,d illustrate the microstructure after hardening heat treatment, which is a mixture of pearlite, bainite and acicular ferrite. In the case of hardening heat treatment cycle, the sample was exposed to an extremely high cooling rate (water quenching), resulting in the formation of the meta-stable bainite and acicular ferrite phases besides the lamellar pearlite phase. It should be mentioned that similar to the scenario of the normalizing heat treatment, the hardening heat treatment also resulted in the formation of a homogeneous microstructure with a uniform grain size throughout the sample from the bottom to the top of the WAAM-fabricated wall. The formation of the acicular ferrite and bainite phases by quenching of the sample at higher cooling rates from the austenite stability region has also been reported in other low-carbon low-alloy steels, such as API X70 and X80 [26,27,35].

### 3.2. Mechanical Properties Evaluation

**Figure 4:**
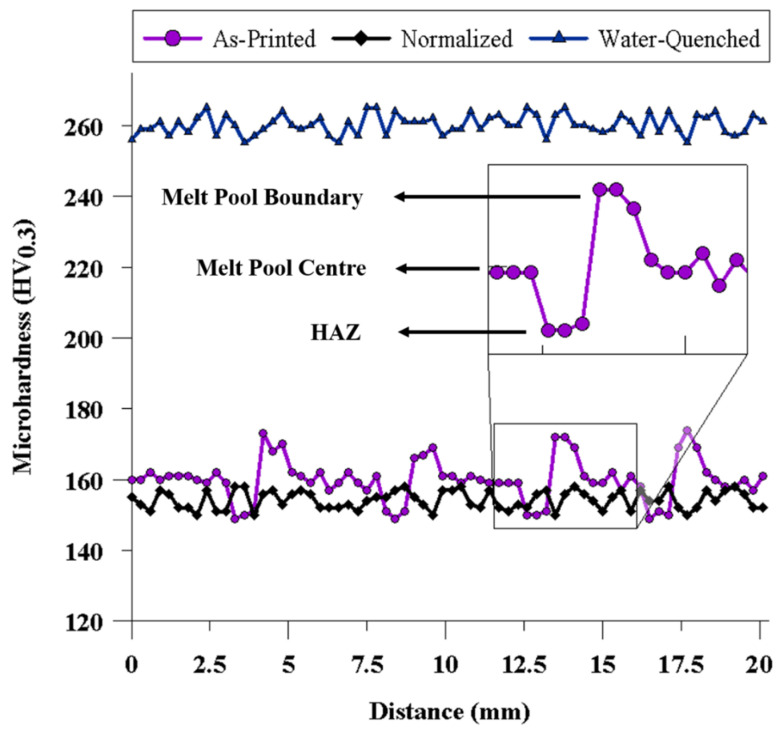
Vickers microhardness profile of the as-printed and heat-treated samples along a line covering five successive layers through the building (vertical) direction.

Figure 4 shows the microhardness profile of the as-printed and heat-treated samples along a line covering five consecutive layers through the building (vertical) direction on different zones, including the center of the melt pools, fusion boundaries, and heat affected zones (HAZs). The overall microhardness of the as-printed sample was 160 ± 7 HV, which showed a relatively significant fluctuation from a minimum of 150 ± 1 HV to 160 ± 2 HV, and then to the maximum of 175 ± 2 HV. The observed fluctuation was ascribed to the presence of different phases along the melt pool center, the fusion boundary, and the heat affected zone. In particular, the lowest amount of microhardness (150 ± 1 HV) corresponded to the HAZ containing coarser polygonal ferrite grains than the rest of the fusion zone, and the maximum microhardness (175 ± 2 HV) was correlated to the fusion boundaries, where acicular ferrite and bainite constituents exist. The center of the melt pool, owning the dominant microstructure of the component (lamellar pearlite and polygonal ferrite), revealed the microhardness of 160 ± 2 HV. The fluctuations in the microhardness values were considerably lower in both hardened (water-quenched) and normalized samples as compared to the as-printed component due to the homogeneity of the microstructure in the heat-treated samples.

The normalized sample with a microstructure analogous to the dominant microstructure of the as-printed sample showed the microhardness of 154 ± 1 HV, comparable to that of the center of the melt pools in the as-printed sample. It is also worth noting that the microhardness of the normalized sample was slightly decreased after the heat treatment, primarily due to (i) the stress relieving occurred during heating to austenitizing temperature, (ii) diminishing of lattice defects formed during the rapid solidification associated with the WAAM, (iii) potential growth of primary austenite grains [36], and (iv) omitting the acicular ferrite and bainite phases from the microstructure of the fusion boundaries. Contrarily, the microhardness of the hardened (water-quenched) sample was 260 ± 3 HV, drastically higher than the other samples, a phenomena ascribed to its microstructure, including pearlite, bainite, and acicular ferrite as the predominant micro-constituents in its structure. It has been reported that the presence of bainite, along with a finer microstructure, can increase the microhardness of low-carbon steels [36]. However, it should also be noted that a higher microhardness is not always beneficial to the overall mechanical performance of the material since the ductility and toughness of the alloy could potentially be degraded. The adverse effect of existing hard micro-constituents can be more crucial particularly in the case of samples manufactured by a welding process, which are usually prone to the presence of welding defects, discontinuities, and residual stresses. Such discontinuities can readily propagate into a brittle microstructure and form internal micro-cracks during the tensile loading of the sample.

**Figure 5:**
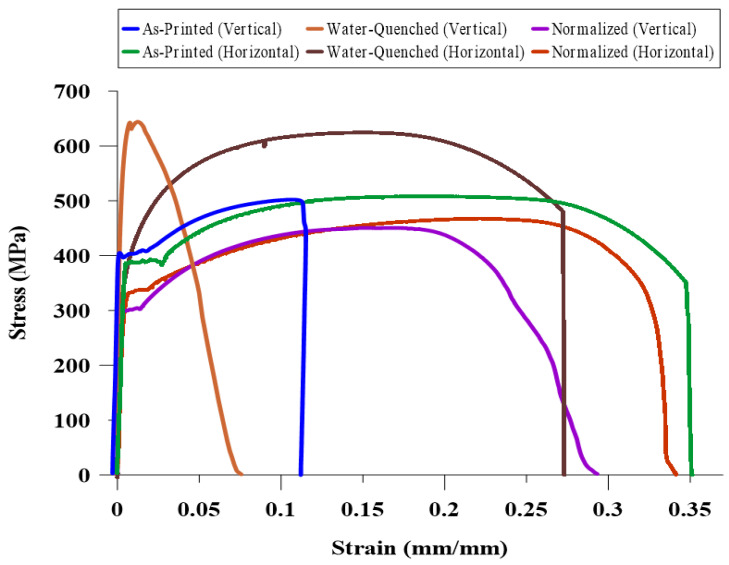
The stress–strain curves for the as-printed and heat-treated samples in the building (vertical) and deposition (horizontal) directions.

Figure 5 shows the engineering tensile stress–strain curves for the as-printed and heat-treated WAAM-ER70S-6 samples in the building (vertical) and deposition (horizontal) directions. In the as-printed component, the vertical and horizontal tensile strengths were approximately similar (~500 MPa). However, the ductility (elongation percentage) of the vertical sample only reached to 12 ± 3%, whereas the horizontal sample showed a significantly higher ductility at 35 ± 2%, indicating a large plastic deformation prior to the fracture with an obvious necking phenomenon. Therefore, the results of tensile testing of the as-printed part revealed anisotropy in ductility. Wang et al. [37] investigated the anisotropy in the mechanical properties of the additively manufactured 304L stainless steel parts, and reported that the elongation percentage in the transverse direction was higher compared to the longitudinal direction, while the tensile strength was fairly isotropic. The substantial lower ductility of the vertical samples as compared to the horizontal ones herein can be justified by (i) the existence of solidification imperfections and flaws, such as inter-pass lack of fusion (LOF) and (ii) the HAZ softening as a result of grain coarsening. Since the long axis of the inter-pass lack of fusions is perpendicular to the loading direction in the vertical samples, the sharp edges of these defects can serve as stress concentration sites, causing the propagation of the discontinuity in the vertical samples during uniaxial tensile loading, but not in the horizontal ones [14]. A similar observation was also reported by Wang et al. [38]. In another study, Lopez et al. [16] demonstrated the formation of manufacturing defects in the wire arc additive manufacturing of ER70S-6 using different nondestructive examination methods including radiographic testing (X-ray), liquid penetrant inspection (LPI), and ultrasonic testing (UT), and reported the presence of the LOF defect between the deposited layers.

The anisotropy in the mechanical properties has been reported as a common issue in various metals and alloys produced by additive manufacturing processes [39,40,41], which can be minimized by applying proper post-printing heat treatment cycles [4,20]. Hardening treatment could increase the tensile strength of the WAAM-ER70S steel from ~ 500 MPa for the as-printed sample to 640 ± 14 MPa and 624 ± 13 MPa in vertical and horizontal heat-treated samples, respectively. This improvement in the tensile strength of the hardened alloy is attributed to its bainitic, acicular ferritic, and pearlitic microstructure with ~ 62% higher microhardness compared to the as-printed sample. However, the ductility of the hardened samples was reduced by 4% and 7% for the vertical and horizontal samples, respectively. Although, the horizontal sample with the ultimate tensile strength (UTS) value of 624 ± 13 MPa and elongation of 28 ± 2% plausibly satisfies the mechanical integrity requirement for the service conditions of this alloy, the anisotropic mechanical behavior of the component cannot be diminished since the sample revealed a severe brittle fracture in the vertical direction with only 8 ± 1% elongation. It should be mentioned that in the case of the as-printed vertical sample, the formation of some defects, flaws, and also possible weak metallurgical bonding between the layers (lack of fusion) can potentially reduce the degree of plastic deformation that the material can accommodate before its failure. This scenario can be intensified when the microhardness increases from 160 ± 7 HV for the as-printed sample to 260 ± 3 HV for the hardened sample. Consequently, as a result of the hardening cycle, the ultimate tensile strength increased at the expense of a reduction in ductility. Overall, it can be inferred that the hardening heat treatment exhibited a positive effect on the mechanical properties in the horizontal direction but was not found beneficial to the vertical sample, leading to its brittle fracture during the uniaxial tensile loading.

On the other hand, the normalizing process that contributed to the formation of a homogenized microstructure, characterized by a uniform grain size along the center of the melt pool, fusion boundary, and the heat affected zone could increase the tensile strength neither in horizontal nor in the vertical samples. Similar to the slight reduction in the microhardness of the normalized sample, its tensile strength was expected to be slightly lower (~465 MPa) than the as-printed sample (~500 MPa). Moreover, a closer look at the stress–strain curves of the normalized samples revealed that there is not a huge difference between the elongation of the component in the vertical (29 ± 2%) and horizontal (34 ± 3%) directions. It should be mentioned that the purpose of the normalizing cycle was to eliminate the inhomogeneous microstructure that resulted from the complex thermal cycles associated with the layer-by-layer deposition nature of the wire arc additive manufacturing process. As a consequence of heating the sample up to a temperature above the upper critical temperature (*Ac_3_*), and formation of new austenite grains, the inhomogeneous microstructure including coarse grains of HAZ was totally eliminated. Accordingly, during the cooling process in still-air, the whole part experiences a similar cooling rate leading to a uniform microstructure at different zones of the sample. Therefore, the anisotropy in the elongation can be eliminated or weaken through modifying the microstructure from an inhomogeneous one to a homogenized microstructure with a uniform grain size.

**Figure 6:**
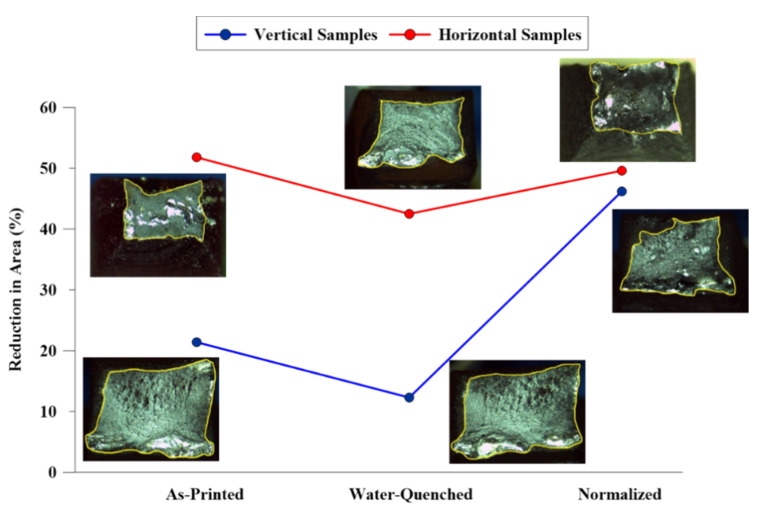
The reduction in area (RA) for the as-printed and heat-treated samples in both vertical and horizontal directions.

Figure 6 demonstrates the reduction in area (RA) for the as-printed and heat-treated samples in both vertical and horizontal directions. The stereomicroscope images of the fractured surfaces are also attached to each point of the plot in order to clarify the brittle or ductile nature of the fracture in different samples. The results of fractography investigations revealed that horizontal samples experienced an entire ductile fracture, while the vertical specimens showed a mixed mode of ductile-brittle fracture, which is consistent with the results obtained from the uniaxial tensile testing (see Figure 5). As clearly shown in Figure 6, there is a considerable difference between the RA values of the horizontal and vertical tensile samples in both as-printed and hardened conditions, implying a significant anisotropy in ductility of the component. However, the RA values of the normalized sample in the vertical and horizontal directions are relatively close to each other, indicating a negligible anisotropy in ductility. Therefore, normalizing treatment can be utilized as a post-printing cycle to minimize the anisotropic behavior of the wire arc additively manufactured low-carbon low-alloy steel (ER70S-6) by homogenizing the grain size and eliminating the inhomogeneous microstructure through the melt pool center, fusion boundary, and the heat affected zone.

## 4. Conclusions

This study aimed to investigate the effect of two post-printing heat treatment cycles, including normalizing and hardening, on the microstructure and mechanical properties of a wire arc additively manufactured low-carbon low-alloy steel (ER70S-6). The following are the key conclusions of the study:

1. The dominant microstructure of the as-printed WAAM-ER70S-6 component consisted of polygonal ferrite grains along with a small volume fraction of lamellar pearlite formed at the ferrite grain boundaries. In addition, the formation of acicular ferrite and bainite constituents were detected as the primary phases along the melt pool boundaries. Furthermore, a heat affected zone comprised of coarser polygonal ferrite grains adjacent to each deposited track in the previously solidified bead also formed, associated with the layer-by-layer deposition nature of the process, inducing multiple heating cycles on each deposited track.
2. Normalizing heat treatment eliminated the meta-stable constituents, i.e., acicular ferrite and bainite, from the as-printed microstructure, leading to a more uniform and homogeneous ferritic/pearlitic microstructure within the melt pool center, fusion boundaries, and the heat affected zone. On the contrary, the hardening heat treatment altered the microstructure of the as-printed part to a combination of pearlite, bainite, and acicular ferrite.
3. Microhardness of the as-printed sample slightly decreased from 160 ± 7 HV to 154 ± 1 HV after the normalizing heat treatment, while the hardening treatment could increase the microhardness to 260 ± 3 HV.
4. Uniaxial tensile testing of the as-printed samples indicated a comparable tensile strength in horizontal and vertical samples, while a considerable anisotropy in the ductility with 35 ± 2% and 12 ± 3% of elongation, in horizontal and vertical directions, respectively, was apparent.
5. Although the hardening heat treatment could increase the tensile strength of the component by around 20%, it intensified the anisotropy in the ductility of vertical and horizontal samples.
6. The anisotropy in ductility was minimized by normalizing heat treatment due to the removal of the coarse grain regions in the HAZ and the resultant uniformity and homogeneity of the microstructure.

## Figures and Tables

**Table 1:** The nominal chemical composition of the ER70S-6 feedstock wire (wt.%).

C	Mn	Si	S	P	Cr	Ni	Mo	V	Cu	Fe
0.06–0.15	1.40–1.85	0.80–1.15	0.04 max	0.03 max	0.15 max	0.15 max	0.15 max	0.03 max	0.5 max	Bal.

**Table 2:** The processing parameters used for the wire arc additive manufacturing of the low-carbon low-alloy steel (ER70S-6).

Average Arc Current	Arc Voltage	Wire Feeding Rate	Scanning Rate	Argon Flow Rate	Heat Input
135 A	28 V	104 mm/s	5 mm/s	20 L/min	7.56 kJ/cm
